# The mental health and psychosocial impact of the Bougainville Crisis: a synthesis of available information

**DOI:** 10.1186/s13033-016-0054-x

**Published:** 2016-03-03

**Authors:** David Tierney, Paul Bolton, Barnabas Matanu, Lorraine Garasu, Essah Barnabas, Derrick Silove

**Affiliations:** St. John of God Frankston Rehabilitation Hospital, 255-265 Cranbourne Rd, Frankston, VIC Australia; Center for Refugee and Disaster Response, Department of International Health, Johns Hopkins Bloomberg School of Public Health, Baltimore, MD USA; Buka Hospital, Autonomous Region of Bougainville, Buka, Papua New Guinea; Nazareth Treatment Centre, Chabi, Autonomous Region of Bougainville, Buka, Papua New Guinea; Buka Hospital, Autonomous Region of Bougainville, Buka, Papua New Guinea; Psychiatry Research and Teaching Unit, Academic Mental Health Unit, University of New South Wales, Southwest Sydney Local Health District, Sydney, Australia

**Keywords:** Bougainville Crisis, Civil war, Mental health and psychosocial impact

## Abstract

**Background:**

The Bougainville Crisis (1988–1997) was the largest armed conflict in the Pacific since WW-II. Despite this, there has been no assessment of the Mental Health and Psychosocial (MHPS) impact of the war. The aim of this paper is to summarize the available data regarding the longer-term MHPS impact of the Bougainville Crisis.

**Methods:**

A literature review and a sequence of consultations in Bougainville were conducted to identify the MHPS impact of the Bougainville Crisis and the capacity within Bougainville to address these issues.

**Results:**

The Bougainville Crisis resulted in violence-related deaths; the displacement of more than half of the population; widespread human rights abuses; far-reaching societal impacts including undermining of the traditional authority of elders and women and damage to cultural values and relationships; property damage; and significant impacts on education and the economy. Conflict-related experiences continue to impact on mental health in the form of trauma-related symptoms, anger, complicated grief, alcohol and substance abuse, domestic violence including sexual assault, excessive alcohol use and a lack of engagement in purposeful activities. Other impacts include an increase in other forms of gender-based violence (including sexual assault), population displacement, and adverse trans-generational effects on children exposed to disturbed parental behaviours attributable to conflict exposure. In spite of the evident needs, there is limited capacity within Bougainville to address these pressing MHPS issues.

**Conclusions:**

The Bougainville Crisis has had a significant MHPS impact at multiple levels in the society. There is a strong interest within Bougainville to draw on external expertise to build local capacity to address MHPS issues. Preliminary recommendations are made to assist the process of building the capacity in Bougainville to address MHPS needs.

## Background

The Bougainville Crisis refers to a civil war that occurred between 1988 and 1997 in the North Solomons Province of Papua New Guinea (PNG), now known as the Autonomous Region of Bougainville. The war, which was the largest armed conflict in the Pacific since WW-II [1–3] was accompanied by widespread human rights abuses and resulted in a significant number of combat and civilian deaths, the displacement (mostly internal) of more than half of the population, and far-reaching social, economic and educational impacts on the society as a whole. Despite the gravity of the upheaval, no thorough assessment has been made of the MHPS impact of the war on the community as a whole.

In October 2009, the first author (DT) was invited by a Bougainville political leader and senior Catholic Nun to engage with local agencies and government on how to address the medium-term MHPS effects of the war. The first author undertook a series of visits and consultations and an extensive literature review on the MHPS impact of the war. The data presented here represents a distillation of first-hand experiences gained during successive visits together with evidence gleaned from existing records and documents. Based on these sources, we review briefly the history of the conflict pertaining to the MHPS impact of the war, and our direct observations from community consultations to reflect on the level of local interest in addressing MHPS issues and the capacity within Bougainville to mount an effective response. We conclude by making preliminary recommendations aimed at assisting in building the capacity in Bougainville to address MHPS needs. In undertaking this task, our explicit intention is to avoid the language of blame in relation to the causes or consequences of the conflict.

### Brief history of the Bougainville Crisis

We provide only a synopsis of the origins and course of the Bougainville Crisis. The complex mix of ethnic, cultural and economic issues that motivated the conflict has been considered elsewhere [5–7]. In November, 1988, landowners with a range of longstanding grievances associated with the operation of the Panguna mine in central Bougainville, damaged mine property, interrupting production and subsequently resulting in its closure in May 1989 [3, 4, 6]. The mine had provided 45 % of PNG’s export income and 12 % of PNG’s gross domestic product [7]. Armed conflict was triggered in December 1988, when the PNG Police mobile squads and later the Police Defence Force (April 1989) were deployed to the area to facilitate the re-opening of the mine [8]. The police tactics used were regarded as harsh by the indigenous people, igniting longstanding secessionist sentiments in Bougainville [6, 9] which in turn garnered broader support for the protest group who subsequently formed the Bougainville Republican Army (BRA), leading to the initially localized conflict spreading across the province [2].

Failure of PNG police to halt the BRA and growing criticism about its handling of the situation compelled the PNG government to accept a ceasefire in March 1990 [10]. Subsequently, all PNG forces and services were withdrawn from Bougainville [5, 10]. In May 1990, the PNG government imposed a total sea and air blockade on Bougainville, preventing the transportation of all goods into the territory including medical supplies [5, 6]. In the absence of PNG Forces, undisciplined factions of the BRA targeted specific groups within Bougainville (the wealthy, well-educated, senior government officers and those suspected of co-operating with the PNG government), resulting in an escalating internecine conflict fuelled by pre-existing feuds and land disputes, in some instances, resulting in frankly criminal acts [3, 4, 6, 8].

In response to a request by Bougainville leaders concerned about the ongoing violence, in September 1990, the PNG Defence Force returned to the administrative centre, Buka Island, to restore and maintain the security and safety of villagers [5]. In July, 1992, the newly elected PNG government took a more assertive position to re-establish control of other parts of Bougainville [8]. In the period 1992–1993, the Bougainville Resistance Forces (BRF) formed in opposition to the BRA, aligning with and supported by the PNG Defence Force [4, 8]. It has been argued that the BRF “bore the brunt of the fighting” on the PNG government side [2]. Armed conflict continued through to the commencement of the peace process in July 1997, and conflict, deaths and injuries continued [6] until February 1999, when the cease-fire agreement was signed, followed in April 2001 by the adoption of the comprehensive Bougainville Peace Agreement [4, 8]. The agreement established Bougainville as an autonomous region within PNG, embodying a commitment to holding a referendum on independence for the territory at some point between 2015 and 2020 [11].

## Methods

We applied two approaches to assess the longer-term MHPS impact of the Bougainville. First, a review of the literature was conducted to identify the conflict-related events that were likely to have impacted on the longer-term MHPS of the Bougainville population. DT searched key databases including MEDLINE, EBSCO Psychology and Behavioural Sciences Collection, PsycINFO, SocINDEX, PsycARTICLES and internet search engines (Google, Yahoo) using the following terms: Bougainville, Bougainville Crisis, Bougainville war AND psychology, psychiatry, mental health, trauma and impact. While no systematic investigations of the MHPS impact of the war were found, various reports documented events that occurred during the war that, according to past research and knowledge from similarly conflict-affected countries were deemed likely to have had a MHPS impact. Reports referred to herein were compiled by official investigators, academics and others who we judged had applied investigative rigour and impartiality to their assessments. Credibility was judged by the identification of sources, accurate specification of dates and locations, and adequacy of the detail given, for example, in identifying the key groups involved (BRA, PNG, BRF). Reports that were based primarily on opinion or appeared to be advocating for a particular side in the conflict were excluded.

Second, during three successive field visits (2009, 2011, 2013), DT made a detailed inquiry into the extent of exposure of the population to conflict-related events, community perceptions regarding current MHPS issues, the level of interest amongst a wide range of stakeholders to address MHPS issues, and the capacity within Bougainville to address these needs. During the 2013 field visit, the Bougainville Mental Health Steering Committee, comprised of local stakeholders, was established to pursue preliminary steps to building capacity in Bougainville in order to address MHPS issues. Subsequently, in 2014, DT, DS and PB were contracted by Counterpart International (an international not for profit organization) with support from USAID to work with the Bougainville Mental Health Steering Committee (including BM, LG, EB) to develop a strategic framework to address MHPS issues. Table 1 provides an overview of the community consultation activities conducted during the three field trips (2009, 2011, 2013) and the joint consultation (2014).

**Table 1:** Overview of community consultation activities regarding MHPS issues

Year	Activities
2009^a^	Meetings with workers from the Nazareth Centre and Leitana Nehan Women’s Development Agency, two non-government agencies attempting to address MHPS issues
2011^a^	Forty people volunteered to meet with DT following an announcement made at a church service about his interest in understanding people’s experiences during and since the war. With the participant’s permission, DT documented thematic information to validate the events likely to have caused MHPS problems (Table 2). No personally identifying information was collected Multiple meetings with workers (n = 7) from three agencies to clarify: data obtained from the literature review (Table 2); the MHPS issues identified in meetings; and the capacity in Bougainville to address MHPS issues
2013^a^	80 people were consulted in a series of meetings and/or at a public forum. Those consulted were President Momis, Ministers and Members of Parliament (n = 4), Senior Parliament/Ministry Officials (n = 3), senior public servants (n = 9), public servants representing departments (n = 8), representatives of women’s groups (n = 7), representatives of church groups (n = 9), village representatives (n = 33), senior staff of three aid agencies (n = 4) and volunteers (n = 2) A public forum was held at Buka Hospital, during which data regarding the events that occurred during the war thought likely to have had a MHPS impact was presented (Table 2). Participants (n = 28) provided feedback and clarification about the direct and indirect MHPS impacts of the war and the capacity in Bougainville to address these issues. An outcome of the public forum was the establishment of the Bougainville Mental Health Steering Committee
2014^b^	DT, DS and PB together with the Bougainville Mental Health Steering Committee (including BM, LG, EB) developed a strategic framework for addressing MHPS issues in Bougainville. This process clarified the current MHPS issues, explored the existing capacity and the potential within Bougainville to address the MHPS challenges arising from the war, and involved 22 meetings, including with President Momis, senior public servants, representatives of women’s groups, workers from agencies attempting to address MHPS issues and aid agencies. A strategic framework to address MHPS issues was submitted to the Autonomous Bougainville Government for review [12]

^a^ Meetings were facilitated by the Bougainville political leader and senior Catholic Nun who initially requested assistance. Meetings in subsequent field trips (2011, 2013) were facilitated by these and other contacts

^b^ Meetings were arranged by the Bougainville Mental Health Steering Committee and other contacts made during the previous field trips

## Results

### Findings: literature review

Table 2 provides a summary of the events that occurred during the war identified by the literature review and likely to contribute to ongoing MHPS issues. Estimates of the number of war deaths vary considerably, but a figure of between 15,000 and 20,000 appears to be most widely accepted in Bougainville. Most deaths involved civilians. Other events relevant to MHPS were the displacement of more than half of Bougainville’s population; the perpetration of wide-scale human rights abuses; undermining of the traditional authority of elders and women, cultural values and relationships; property damage; and major disruptions to education and the economy. Participants engaged in the series of consultations during field visits affirmed the importance and widespread nature of the conflict-related events listed in Table 2. The consensus view, however, was that the data gleaned from the literature review underestimated the scale and gravity of the events that occurred during war and their likely contribution to ongoing MHPS issues [14].

**Table 2:** Summary of war events that are likely causes of MHPS problems

Event(s)	Evidence/comment
Total deaths attributed to the war	Estimates of the total number of deaths vary from 12,000 [13] to 20,000 [2] from a pre-war population of 160,000 [4] In 1993, half way through the war, the Minister of Health had compiled the names of 10,000 people who had died [14]
Deaths due to deprivation of medical supplies	Incomplete data from 18 of 23 Health Centres reported 2023 deaths from normally preventable diseases in the period January 1990–July 1991 [15]^a^ The blockade of all goods including medical supplies May 1990–September 1994 [16] in areas controlled by the PNG Defence Force, but lasted up to 8 years in areas controlled by the BRA [17] Many of the 149 health facilities were destroyed and many health workers displaced [13]. Between 1992 and 1998, there were no doctors in central and southern Bougainville for a population of approximately 100,000 people [1]
Combat related deaths	Estimates vary from 1000–2000 deaths for the whole war [4, 14] to 1500 deaths over about 26 months [10] ^b^. There are reports of bodies buried in mass graves and dumped at sea from helicopters [14]
Extra-judicial killings murders and disappearances	Investigators confirmed 158 extra-judicial killings and 13 disappearances [10, 18] ^b^, but concluded that the number of extra judicial killings was “undoubtedly higher” ([18], p. 1)^b^ The alleged extra judicial killings of 374 people and the murder of 166 people was reported [15, 19]^a^. Additionally, there are reports of extra-judicial killings where the number killed is unknown (e.g. “a group” [15], p. 12^a^). It is unclear if these numbers are additional to or inclusive of those in other reports [10, 18]^b^
Displacement of population	More than half of the population was displaced: 15,000–20,000 fled Bougainville [4]; 67,300 were internally displaced into care centres (internal refugee camps) [18] and nearly half of those in these centres were under 15 years of age [1]; and unknown numbers moved to BRA bush camps and hid independently [1]. About 50,000 people were living in care centres at the time of the cease-fire [13] Displacement to care centres included forced relocation following the destruction of villages and the perpetration of human rights abuses within these centres [10, 15, 18, 19]^a, b^
Sexual assault	Reports range from the alleged sexual assault of individuals through to the sexual assault of “many” in care centres, pack rapes, individuals being murdered after being raped, women committing suicide after being raped and people being detained and sexually assaulted over weeks [10, 14, 15, 18–20]^a, b^. While the numbers who were sexually assaulted is unknown, thousands of files of rape victims were destroyed by combatants who feared the implications of these records in relation to possible action regarding war crimes [4]. Reports of sexual assaults contrast with the view of there being a low pre-war incidence of sexual assault due to unique cultural factors in a largely matrilineal society [21, 22]
Deliberate and indiscriminate gunfire	People in villages, boats and canoes were subjected to indiscriminate gunfire from land, patrol boats and helicopters [6, 10, 15, 18–20]^a,b^ 120 reports of alleged incidents: with some resulting in deaths and property damage; that range from discrete short lasting events to those that were sustained for a few weeks; and incidents that appeared to have specific targets to those that covered broad geographical areas [15, 19]^a^
Harassment, beatings and torture	124 reports of alleged incidents affecting individuals through to whole villages [15, 19]^a^ Evidence in other reports [10, 14, 18, 20]^b^
Undermining of traditional authority	The traditional authority of elders and women was undermined by military command [23]
Damage to important values and relationships	Important values and pro-social relationship dynamics were damaged in care centres [24] and “deep divisions” [8, p. 26] were created amongst Bougainvillians who fought against each other [14]
Property damage	118 alleged incidents resulting in the destruction or damage of residential and commercial property, food gardens and crops, theft and the killing of animals [15, 19]^a^. Events documented range from the burning of one home to the destruction of clusters of villages resulting in the displacement of 10,000 people. It is estimated that the homes of one-third of the population were destroyed [4, 14]; 14 million cocoa trees were destroyed [1]; and that only 20 % of cocoa trees remained in Southern Bougainville [1]
The collapse of the education system	Between 15,000 and 20,000 children were denied an education due to the closure and/or damage to schools [1]. Prior to the war Bougainville had the highest standard of secondary school education in PNG [13]. In 2011, a number of schools had not resumed [14]
Economic	The almost total destruction of economy and infrastructure [1, 8, 16] The pre-war economy was “dominated” by the Panguna mine ([1], p. 50). It has been estimated that in 1989 the mine would have contributed 4000 direct and 8000 indirect jobs [25]. The mine has not reopened. Cocoa and copra are Bougainville’s largest export crops [26] and historically have been the major source of income for the majority of the population [25]. In 1988/98, 18,000 tons of cocoa and 27,000 tons copra were produced and this fell to negligible levels during the war [1]. By 2006, cocoa and copra production had increased to 15,000 and 12,800 tons respectively [26]

^a^ Data was collected under considerable duress, and the author noted that the 85-page compilation is likely to underestimate the full extent of human rights abuses. Further, the Bougainville Peace Agreement pardoned all combatants and thus there has been no further investigation of alleged human rights abuses [11]

^b^ Restrictions imposed on investigators and threats made to civilians regarding the provision of information to investigators, suggest that these reports underestimate the incidence of human rights abuses

### Findings: community perceptions of ongoing MHPS issues and the need to address these issues

Table 3 summarizes community perceptions regarding current MHPS issues among specific groups and across the broader community that were consistently identified across a sequence of consultations (2009, 2011, 2013, 2014) and, where available, from research and existing documentation. Absence of epidemiological data, with the exception of information regarding domestic violence and sexual assault [27, 28], limits the extent to which the prevalence of the problems identified in Table 3 can be specified. Nevertheless, in personal communication with DT, Mr. Beleh (local politician) and Sr. Garasu (Catholic Nun) reported that they had identified 232 ex-combatants as manifesting one or more MHPS problems arising from the conflict in one of Bougainville’s 33 political constituencies. Those who were children/adolescents during the war (the lost generation) were reported as having limited education, a lack of engagement in traditional values and activities and displaying aberrant behaviours all of which contribute to their marginalization in the community. Many of this group experienced events during the war likely to have been traumatic [14, 24] and while the numbers who continue to be impacted by these experiences is yet to be determined, an expatriate Marist Brother teacher/counsellor noted that most of 50 male students he taught had been involved in combat and appeared to be suffering from symptoms of post-traumatic stress [24]. Various reports, largely based on community perceptions, have highlighted the detrimental effects of the conflict on subsequent risk of substance abuse [14, 23, 29, 30]. Gender-based violence including sexual violence is considered a significant issue in Bougainville [27, 28] and thought to continue at a higher rate compared to that prior to the war. The number of people missing presumed to have died during the war is unknown, but the inability to conduct customary burial ceremonies was reported as resulting in complicated grief for the surviving relatives and as having broader impacts including land title and use issues [14]. Meetings with senior police personnel highlighted the difficulties the police force experience in carrying out their duties amongst a community impacted by the war and its aftermath [14] and by some officers who continue to be impacted by their personal war experiences. People continue to be displaced since the war and family separation, separation from traditional land, insecure living circumstances and the strain on host communities/families were reported as continuing impacts [14]. Finally, our informants reported a trans-generational impact on those born after the war through their exposure to a range of trauma-related aberrant behaviours displayed by parents and within the community at large.

**Table 3:** Summary of community perceptions about current MHPS issues

MHPS issues^a^	Community perceptions
Ex-combatants	Ex-combatants were described as displaying behaviours thought to reflect the long-term impact of trauma exposure including substance abuse; weakening of family responsibilities; conflict with spouses concerning the use of money to purchase alcohol; perpetration of violence (including sexual) against women and children; and the use of sex as a coping mechanism. Substance abuse and the perpetration of sexual assault by ex-combatants have been reported elsewhere [13, 14]. While the number of ex-combatants displaying these behaviours across Bougainville is unclear, in personal communication with DT, a local politician and Catholic Nun reported that 232 ex-combatants were identified in one of Bougainville’s 33 political constituencies as manifesting one or more of these problems
Lost generation	Those who were children/adolescents during the war are referred to as the “lost generation” in Bougainville. This group were described as being marginalized and alienated, having limited to no formal education, lacking engagement with traditional social values and roles and displaying aberrant behaviours Some of this group continue to be impacted by their war experiences which include armed combat, witnessing human rights abuses and being displaced into care centres [14, 24]. While the number within this group who are adversely impacted by these experiences is unknown, a few years after the war an expatriate Marist Brother teacher/counsellor noted that most of 50 male students, who had been involved in combat, were impacted by post-traumatic stress including displaying a range of aberrant behaviours considered to be trauma related [24]
Substance abuse	Substance abuse (home brewed and commercial alcohol and marijuana) is considered a major problem and was reported to be associated with rape and unwanted pregnancies, fighting, criminal behaviour, the destruction of village values and drug induced psychosis. These detrimental impacts have also been identified in a number of reports [14, 23, 29]. Substance abuse has been linked with unresolved trauma [14, 23]
Gender violence	Gender-based violence including sexual violence is considered a significant issue in Bougainville. The view is that gender-based violence including sexual assault has continued at a higher rate compared to that prior to the war. Qualitative research has identified a high prevalence of gender-based violence including sexual assault in Bougainville [27, 28]. Gender based violence has been linked with unresolved trauma [14]
Missing persons	People continue to search for the remains of relatives, who are presumed to have died during the war, to return them to their clan for customary burial. It was reported that the inability to perform customary burial ceremonies complicates the grieving process and has implications for land ownership and use [14], The numbers impacted by their inability to locate the remains of relatives is unknown, but it was estimated that there are “many” ([14], p. 7). The importance of the issue however is reflected by the fact that in 2014 the Autonomous Government of Bougainville acknowledged the suffering of the relatives of the missing and adopted a policy to clarify their fate [33]
Police force	Senior police reported considerable difficulties for the police force generally coping with working in a post-conflict community impacted by a range of MHPS issues. They also reported that some officers who are ex-combatants continue to be impacted by their war experiences, and this impacts their work performance and families. The various issues contributing to the difficulties policing in Bougainville have been reported elsewhere [14]
Displacement	People continue to be displaced within and outside Bougainville. Some are living with relatives causing great strain on host families, while others are living insecurely squatting on land belonging to others [14]. Reasons reported for not returning to their villages include unresolved trauma, fear of rejection for past crimes and threats of violence [14]. The burdens of displacement include separation from families and traditional lands [14]. The numbers who continue to be displaced is unknown but in 2003, as many as 9000 who fled to the Solomon Islands had not returned [1] and it has been estimated that thousands of families continue to be displaced within Bougainville [14]
Trans-generational impact	A trans-generational impact of the war appears to be emerging among those born after the war. Reports indicate that this group have been impacted by their exposure to a range of trauma-related aberrant behaviours in parents and the society at large (e.g. excessive drinking, weakening of family and community structures, absence of customary guidance and role models previously provided by traditional authority structures)

^a^ Credibility was judged by the consistency MHPS issues were reported across all consultations. Issues that appeared to be pushing a personal or political agenda were excluded

Collectively, the MHPS identified were described as having a broad impact on the social fabric of Bougainville society and, indirectly, on economic recovery. Across the sequence of consultations (2009, 2011, 2013, 2014) there was a broad consensus concerning the priority need to address MHPS issues to reduce the distress experienced by individuals and the burden of associated disturbed behaviours on families, community, health services, the police, the judiciary and educational institutions. The overarching view of our informants was that addressing MHPS issues, particularly trauma related aberrant behaviours had the potential to: assist in healing relationships damaged during and since the conflict; support efforts to maintain and enhance the peace process; and encourage the re-engagement of those affected in purposeful and productive family, community and economic activity. It was recognized that, in addition to the need for MHPS services, traditional reconciliation ceremonies in which perpetrator(s) compensate victims, might play a complementary role in achieving healing for sides of the conflict.

### Findings: current resources to address MHPS issues

While a range of government, non-government (secular, faith-based, voluntary) services, agencies and groups are confronted by MHPS issues in their daily work, only a few agencies are specifically focused on assisting persons with these problems. Two such agencies are the Nazareth Centre, which provides refuge for women and other survivors of family violence, youth who have substance abuse issues and treatment for former combatants; and the Leitana Nehan Women’s Development Agency who provide counselling and referral related to gender based violence, trauma-related awareness raising programs and training for community based organizations.

In addition to the limited number of trained staff to address MHPS issues across Bougainville, there are minimal resources to attend to people with severe mental disorders including those with psychotic disorders (schizophrenia, bipolar disorder, and other psychotic disorders), neuropsychiatric conditions/brain disorders, severe mood disorders and chronic traumatic stress disorders that are typically found in low-income, post-conflict settings [32–34]. Yet at the time of writing, there is only one mental health nurse for the total Bougainville population of 254,000 persons. Patients referred to the national hospital are assessed and treated by the mental health nurse supported by hospital physicians, noting that the hospital is distant from and difficult to access from many areas of Bougainville. The only options for care for the more severely disturbed patients referred to the hospital are short-term accommodation in a centre designed principally to provide refuge for women experiencing domestic violence; being held in police cells with criminal offenders; or being transported by air (with medical and police escort) to the national capital, Port Moresby, for specialist treatment. The inadequacies of the system add credence to observer reports that the mentally ill are at risk of neglect (and in some cases abuse) throughout Bougainville.

This brief overview indicates that while key individuals and agencies have worked valiantly over many years to address MHPS issues, it is evident that the capacity within Bougainville to address these issues is severely limited relative to the demand and this has been noted in a number of reports [14, 23]. The issues identified during the consultations constraining the capacity in Bougainville to address current MHPS issues are in Table 4.

**Table 4:** Issues constraining the capacity in Bougainville to address current MHPS issues

Constraints on the capacity in Bougainville to address MHPS issues
No formal assessment of MHPS needs across Bougainville
Limited access to evidence based training and professional supervision of workers
Limited opportunity to build institutional capacity
Insecure and intermittent funding
Lack of resources and support to build a strong organizational framework to achieve consistent co-ordination and integration of services
Inadequate resources to implement and monitor evidence-based practice, lack of capacity and resources to undertake systematic treatment outcome assessments
The absence of an overarching and integrated plan to advance the mainstreaming of mental health services as an essential component of overall health service development

On the positive side, important facilitating factors were made evident during the consultations regarding the potential to address MHPS issues, as outlined in Table 5. A clear recognition of the need to address MHPS was identified throughout the consultation process as was a strong interest in building Bougainville’s capacity to address these needs and the Government’s interest in developing the necessary supportive policy and legislative framework.

**Table 5:** Facilitating factors to address current MHPS issues

Facilitating factors
Broad recognition of the need to address MHPS issues
Interest in building the local MHPS capacity
The presence of key individuals and agencies interested in being actively involved in addressing MHPS issues
During the 2014 consultancy, President Momis identified the need for the government to take a greater role on caring for those who have mental health problems, including the need for legislation to support this proposal

## Discussion

It is widely accepted within Bougainville that the Crisis left in its wake a range of MHPS issues that require urgent attention. Specific groups have been identified as at high risk of MHPS problems but research in similar situations would suggest a broader community impact. There has also been no assessment of the numbers or needs of those who have severe mental disorders in Bougainville, nor the needs of their families. Although it has been generally assumed that 2 % of any population have a severe mental disorder, findings from a comparable population (Timor-Leste) have found a figure closer to 3 % [33]. In other post-conflict low resource countries up to a third of exposed persons have been found to experience broadly defined traumatic stress reactions [35], and those directly exposed to the more severe traumas of mass conflict (including torture, other forms of human rights abuses, combat, exposure to murder, gender-based violence) have an increased risk of experiencing a range of disorders and reactions including complex PTSD, prolonged grief, explosive anger, somatoform disorders, drug and alcohol problems and psychotic-like reactions (often presenting as culturally specific reactions) [36, 37]. The majority of the Bougainville population has directly experienced war or its aftermath including ongoing violence, stress, and/or physical and economic disruption. Informants in our consultations (2009, 2011, 2013, 2014), supported by relevant research/reports highlighted the following as significant current mental health issues: substance abuse [14, 23, 29]; gender based violence including sexual assault [14, 27, 28]; displacement-related stressors [14]; complicated grief and land title/use issues for relatives who are unable to perform customary burial rituals for missing persons thought to have died during the war [14]. Adverse trans-generational effects amongst those born after the war exposed to a range of trauma-related aberrant behaviours in parents and the society at large was also reported by informants. The widespread erosion of the social and cultural fabric of the society has had negative impacts at a population level, adding to a context of psychosocial vulnerability and hence, risk of mental disorder. At the societal level, undermining of the economy, the capacity of conflict-affected persons to use existing resources and constraints in the institutional framework to address these issues have severely limited the capacity of Bougainville to attend effectively to the broad psychosocial needs of the population as well as to specific mental health and traumatic stress problems experienced by a substantial minority.

## Limitations

There is a lack of systematic and comprehensive data concerning MHPS needs in Bougainville [14]. The data cited in this paper therefore can only provide indicators of need based on broad observations and estimates. The consultation process undertaken did not include the collection of demographic information and it is therefore unclear to what extent the views of the informants is representative of the population. Further, while informants consistently reported that gender based violence, including sexual assault, and substance abuse have continued at a higher rate compared to that prior to the war, there are no pre-war data to allow comparison. There may also be an attributional bias within Bougainville (as in other conflict-affected settings) in assigning responsibility for most current MHPS to the direct or indirect impacts of the war.

### Recommendations

A sequence of consultations (2009, 2011, 2013, 2014) identified that mental and psychosocial problems are a priority in Bougainville, and that there is broad support amongst politicians, senior public servants, service providers and the general community to build Bougainville’s capacity to address these issues. There appears to be a consensus that addressing MHPS issues would reduce the distress experienced by individuals with MHPS problems and the burden experienced by families and others caring for them. Further, unresolved trauma has been associated with a range of aberrant behaviours (e.g. violence including gender based violence and sexual assault, substance abuse) impacting the community at large, as well as a range of specific services (e.g. police, health) and, more broadly, economic activity [14, 23, 24]. It is anticipated that the provision of appropriate treatment and support for those affected will have broad community benefits, including contributing to the ongoing peace process [14].

The following recommendations, developed in close collaboration with the Bougainville Mental Health Steering Committee, have been submitted to the Autonomous Bougainville Government for review [12]. (1) To conduct systematic research regarding MHPS needs, current practice in caring for the mentally ill, and service provider capacity. (2) To use the resulting data to develop a comprehensive mental health model for Bougainville. The model would include three service levels (village-based, community level health service and hospital based mental health service) dependent on an assessment of need and response to treatment, and a referral system between the various levels of service. It could include community based psychosocial support and psychotherapy approaches for PTSD, depression, and anxiety that have been found to be effective in other low resource environments (e.g. [38–40]). The service delivery model would incorporate the building of institutional and worker capacity with strategies and ongoing resources to ensure sustainability and the mainstreaming of mental health services as an essential component of overall health service development. (3) To similarly develop and test strategies to raise awareness and advocacy to highlight mental health within the broad community with the aim of improving knowledge of and support for those with mental health issues and their families. (4) To develop an overarching mental health policy, based on these findings and available literature, with the aim to provide sustained and accessible services to the Bougainville population.

## Conclusion

Although systematic data are lacking, existing information suggests that the majority of people in Bougainville have been significantly affected by the trauma, stress and disruptions resulting from the war. There is an immediate need and a strong interest within Bougainville for assistance to build local capacity to address MHPS issues. This situation warrants assertive and immediate action in relation to the more precise delineation of the mental health status of the Bougainville people and development of comprehensive services to address the multiple needs of the population.
